# Characteristics and Scenarios Projection of Climate Change on the Tibetan Plateau

**DOI:** 10.1155/2013/129793

**Published:** 2013-07-22

**Authors:** Zhenchun Hao, Qin Ju, Weijuan Jiang, Changjun Zhu

**Affiliations:** ^1^State Key Laboratory of Hydrology-Water Resources and Hydraulic Engineering, Hohai University, Nanjing 210098, China; ^2^NAVECO Ltd., Nanjing 210028, China; ^3^College of Urban Construction, Hebei University of Engineering, Handan 056038, China

## Abstract

The Fourth Assessment Report of the Intergovernmental Panel on Climate Change (IPCC AR4) presents twenty-two global climate models (GCMs). In this paper, we evaluate the ability of 22 GCMs to reproduce temperature and precipitation over the Tibetan Plateau by comparing with ground observations for 1961~1900. The results suggest that all the GCMs underestimate surface air temperature and most models overestimate precipitation in most regions on the Tibetan Plateau. Only a few models (each 5 models for precipitation and temperature) appear roughly consistent with the observations in annual temperature and precipitation variations. Comparatively, GFCM21 and CGMR are able to better reproduce the observed annual temperature and precipitation variability over the Tibetan Plateau. Although the scenarios predicted by the GCMs vary greatly, all the models predict consistently increasing trends in temperature and precipitation in most regions in the Tibetan Plateau in the next 90 years. The results suggest that the temperature and precipitation will both increase in all three periods under different scenarios, with scenario A1 increasing the most and scenario A1B increasing the least.

## 1. Introduction

In the context of global warming, climate specialists all over the world show great attention to the projection for future local climate change. Currently, global climate models are considered as an important tool for understanding attributions of past climate change and predicting the future [1–5]. Therefore, it is very important to assess the ability of those models to reproduce the observed climatological features, which will directly affect the “reproduction” of current decadal climate changes, and verify to some extent the credibility for future climate change projections.

IPCC (the Intergovernmental Panel on Climate Change) Data Distribution Center (IPCC-DDC for short) released the Fourth Assessment Report with climate scenarios simulations and projections with 24 climate models. Some climate models predicted climate changes under 9 emission scenarios, among which 3 scenarios are identified as the most important ones for future climate change: high scenario SRES A2 (Special Report on Emission Scenarios A2); middle scenario SRES A1B; and low scenario SRES B1 [1, 2]. It has become a hot research issue to assess global and regional simulation ability and predict climate change tendency for different emission scenarios with a single model or multiple models [3, 4]. Phillips and Gleckr [6] evaluated the ability of the 20 IPCC-AR4 (the Fourth Assessment Report of the Intergovernmental Panel on Climate Change) models to reproduce global land annual mean precipitation; the result shows that new global climate models have better simulations of global land precipitation than the previous version. Based on the atmospheric circulation features of European climate change, AP van Ulden and van Oldenborgh [7] assessed the simulation ability of global climate models over Europe by calculating spatial correlation between observed and simulated values; the result shows that there are 8 models which have well-projected European atmospheric circulation changes. Johnson and Sharma [8] assessed the credibility of global climate models in time and space using the “Variable Convergence Score (VCS)” method. Sun et al. [9] evaluated the ability of new global climate models to reproduce frequency, intensity, and other indicators of precipitation, which indicated that precipitation was still too frequent in the latest models. Chinese scientists have been working on future climate change assessment in China. Zhou and Yu [10] assessed the ability of the 19 IPCC AR4 models to reproduce precipitation climatology in China; the result shows that new GCMs simulated much better than the previous with regard to China's climate; most models could represent the average of surface temperature, but the simulation of warming ratio in recent years still needs to be improved. With the 22 IPCC AR4 models, Xu et al. [11] evaluated ability of various models to reproduce China's climate changes in the 20th century; and then pointed out that the temperature simulation of each model was better than precipitation, while that multimodel ensembles mean simulated much better than a single model. Jiang et al. [12, 13] analyzed the ability of the IPCC-AR4 models to reproduce precipitation, temperature, and extreme precipitation index and project future climate changes under different emission scenarios in China. In addition, a lot of research has been done by scholars on climate change assessments and projections of GCMs [14–17]. 

Messerli and Ives [18] pointed out that mountainous and highland areas were vulnerable and sensitive to climate changes. The climate environments ecosystems in these regions respond most rapidly and significantly to global climate changes [19]. Climate warming on the plateau in past decades has been suggested by meteorological observation and ice core records [20]. The climatic warming trend seems more evident on the plateau than globally during the period from 1950 to 1993 [21]. Therefore, trend analysis of climate changes in high-altitude regions is very important for research on climate changes tendency. Being at an average elevation about 4000 m, the Tibetan Plateau is known as the roof of the world, which covers over 200 million km^2^ with steep terrain. Besides it has far-reaching effects of global climate changes [22]. Although the Tibetan Plateau has a significant impact on atmospheric circulation and hydrological cycle, researches on the Tibetan Plateau climate changes have been few because of lack of data.

## 2. Data Sets and Methodology

**Figure 1:**
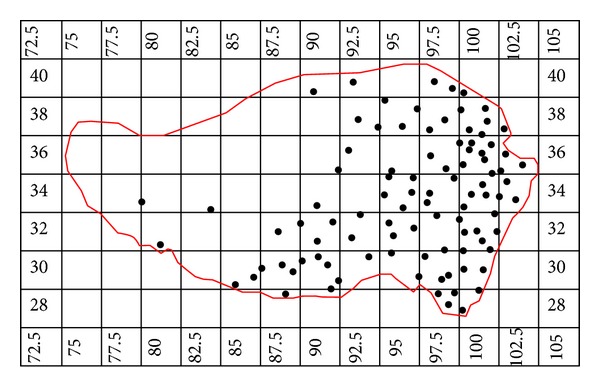
Grid patterns of the GFCM21 climate models on the Tibetan Plateau.

With monthly average temperature and precipitation data of reference period (20 C3 M) of 22 IPCC-AR4 models under contemporary climate condition, this paper compares these data with observed values from 96 meteorological stations on the Tibetan Plateau (Table 2) and assesses generally the ability of GCMs to reproduce precipitation and temperature climatology on the Tibetan Plateau and climate projections under different emission scenarios, in order to make deeper understanding of China's climate changes in the context of sustaining global warming and provide basis for assessing impacts of climate changes on hydrology and water resources.  Table 1 lists the basic information about 22 GCMs, as well as the border and number of grids of the Tibetan Plateau. More detailed information about GCMs can be found at http://www-pcmdi.llnl.gov/ipcc/about_ipcc.php. Take GFCM21 pattern from American Geophysical Fluid Dynamics Laboratory, for example. Distribution of grids on the Tibetan Plateau is shown in Figure 1; small black dots indicate 96 meteorological stations.

Currently, the method of multimodel ensembles mean [10, 12, 23] is commonly used for analyzing global climate models data. Compared with a single mode, multi-model ensembles mean reduces errors and reproduces- a more realistic situation. However, with calculating the arithmetic average of multi-model data, characteristics of each model are disappearing in this method, which is not conducive to distinguish simulation features from each model. In order to assess the ability of each model to reproduce temperature and precipitation on the Tibetan Plateau, based on original grid data of monthly temperature and precipitation from GCMs, we have calculated regional monthly average of reference period for each model, analyzed differences between simulated and observed values in time and spatial scales, and taken relative error, absolute error, correlation coefficient, and deterministic coefficient as performance indeices of GCMs [24].

## 3. Model Evaluation: 20th Century Climate of the Tibetan Plateau

### 3.1. Temperature

As seen in Table 2, from comparison between simulations and monthly average observations of 96 stations on the Tibetan Plateau of reference period (1961 ~ 1990), the observation and the simulated values are highly relevant while most correlation coefficients are above 0.96 except INCM3 pattern. So, GCMs have well-simulated temperature on the Tibetan Plateau in a certain degree. The annual mean temperature of the Tibetan Plateau during reference period is 3.3°C. There are great differences between models in simulating multiyear temperatures, while all simulated temperatures are lower than observed values. Compared to observed values, BCM2 pattern has the largest absolute error, that is, 11.6°C below observation; INGSXG pattern has the smallest absolute error, that is, 3.4°C below observation. With regard to deterministic coefficient, only a few patterns have relatively good simulation ability. 

**Figure 2:**
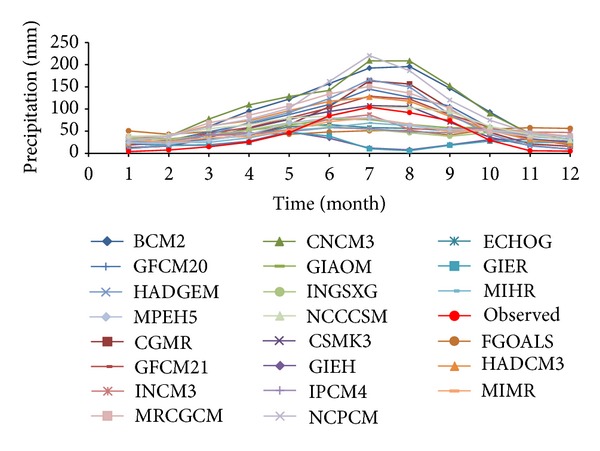
Comparison of annual temperatures (1961 ~ 1990) on the Tibetan Plateau between simulation and observation.

**Figure 3:**
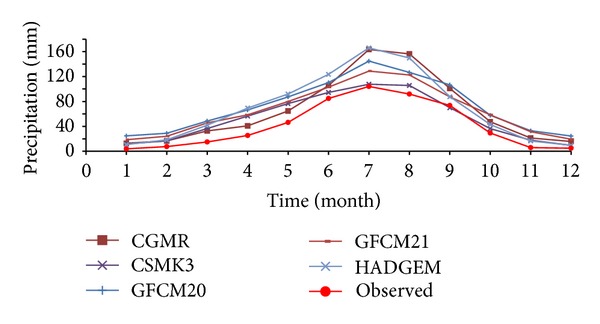
Comparison of five best-performance climate models for temperature.

With regard to comparison between multiyear monthly mean temperature in reference period of various models and the observed data (Figure 2), it shows that the simulated values of all modes are smaller than the observed; however, the high ones (monthly mean temperature from June to August) are floating around observation. We selected five patterns with the best simulation ability (Figure 3): GGMR, GFCM21, HADCM3, HADGEM, and MRCGCM. Figure 3 shows that climate changes with five models are consistent with the fact, which is also confirmed by Table 1. As seen from Table 1, these five models own a high value of uncertainty coefficient and the best simulation ability in annual mean temperature. 

**Figure 4:**
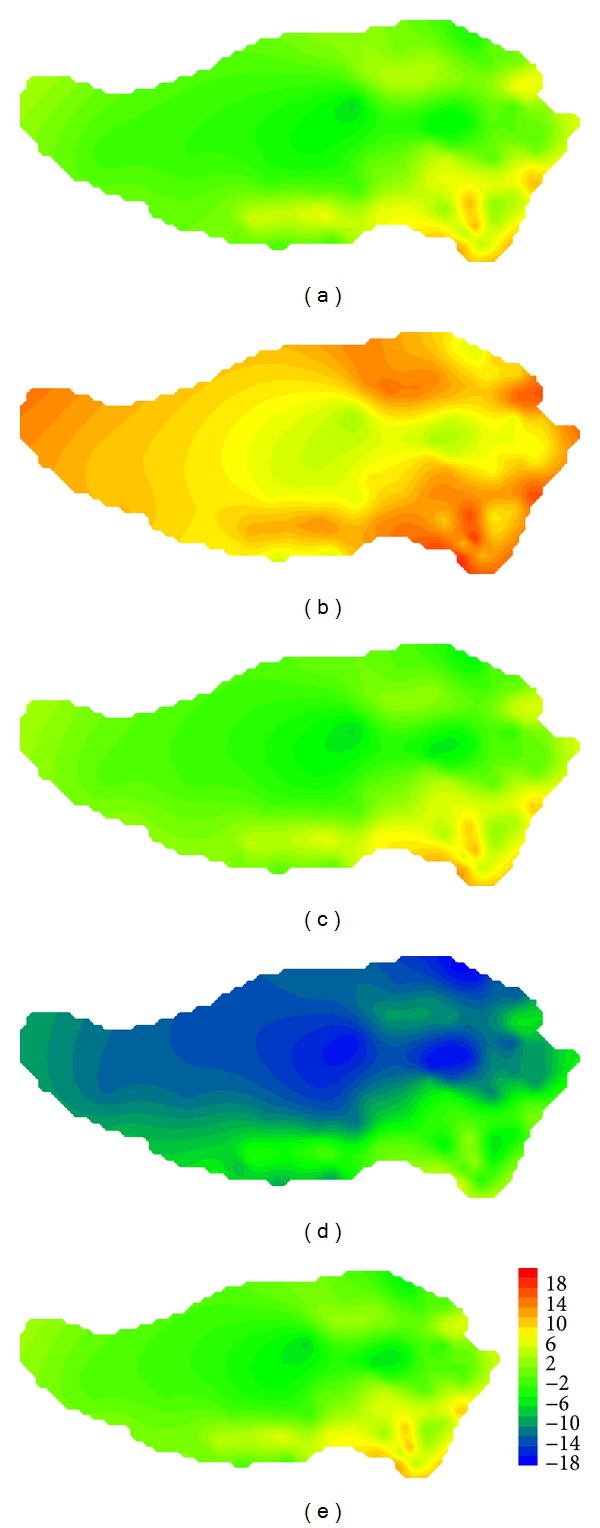
Observed values of temperature on the Tibetan Plateau in reference period: (a) spring; (b) summer; (c) fall; (d) winter; (e) annual.

**Figure 5:**
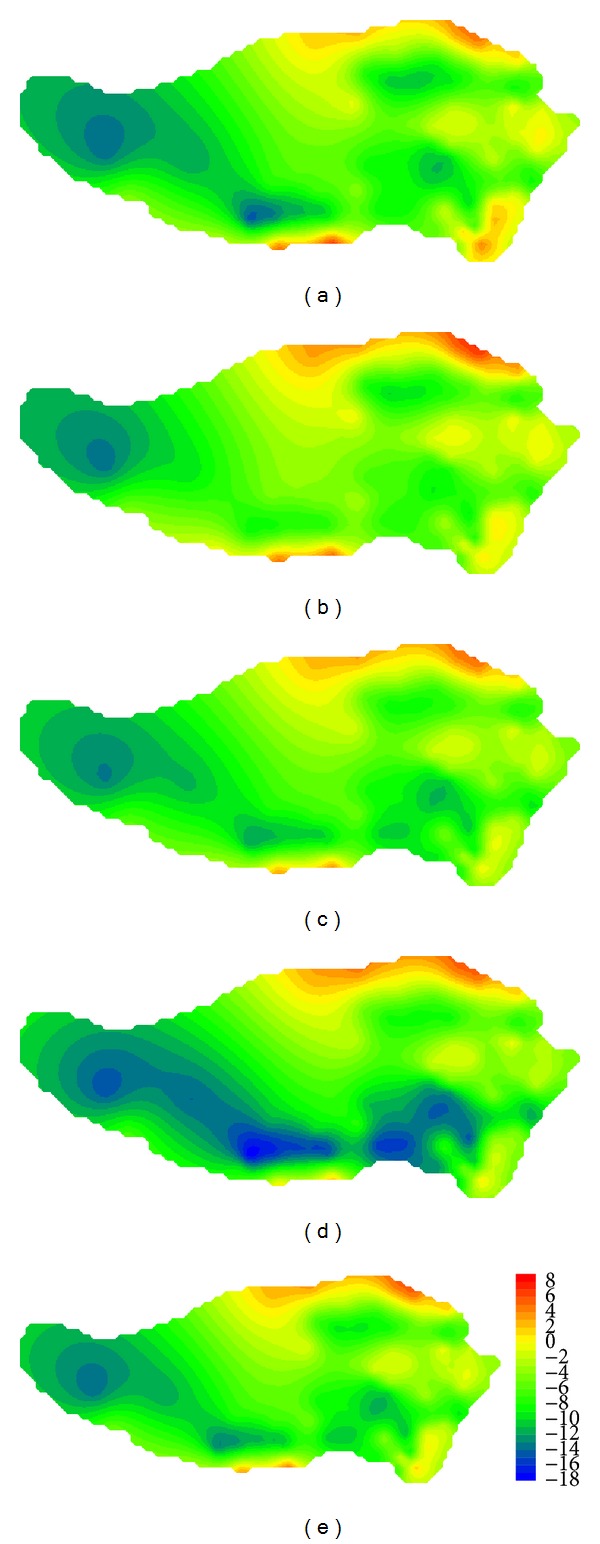
Comparison of the values of temperature on the Tibetan Plateau in reference period between GFCM21's simulation and observation: (a) spring; (b) summer; (c) fall; (d) winter; (e) annual.

Based on grid output of each model, we have interpolated seasonal mean temperature for each station with Delta-DCSI downscaling method [25]. Traditionally, spring is from March to May; summer is from June to August; fall is from September to November; winter is from December to February of next year.  Figure 4 shows the observed values of temperature on the Tibetan Plateau in reference period, which indicates that the average temperature of the Tibetan Plateau is between −5.6 and 14.5°C, with significant seasonal and spatial differences: high temperature in the southeast, decreasing from south to north and from east to west. Take GFCM21 as an example; we have analyzed differences between simulated and observed values of temperature in reference period (shown in Figure 5). Figure 5 shows that simulated temperature of GFCM21 pattern, which has a deviation of −18.1°C ~ 7.2°C from the observed, is much lower than the observed in most part of the region except for edge of the northern and southern areas. Annual mean temperature has a deviation of −14.0°C ~ 5.4°C. The maximum deviation has met −18.1°C which occurred in the western region in fall. The deviation was decreasing from west to east.

### 3.2. Precipitation

The same method has been applied to assess the ability of each model to reproduce precipitation. Annual mean precipitation on the Tibetan Plateau was 492.5 mm in reference period which was lower than simulated values of most models (Table 2). Different models had great differences in simulation ability while more than half of models had bad simulation performance. The maximum relative error is up to 155.8% with CNCM3, while the minimum is 6.0% with IPCM4. Some models have good correlation coefficient between simulated and observed values. There are more differences in precipitation simulation ability than in temperature of each model.

**Figure 6:**
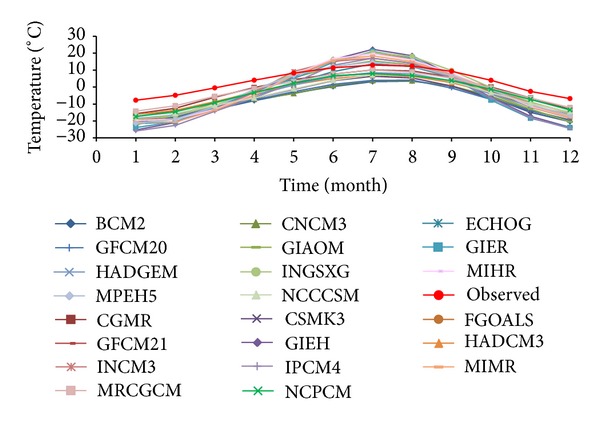
Comparison of precipitation on the Tibetan Plateau between simulation and observation in reference period (1961–1990).

**Figure 7:**
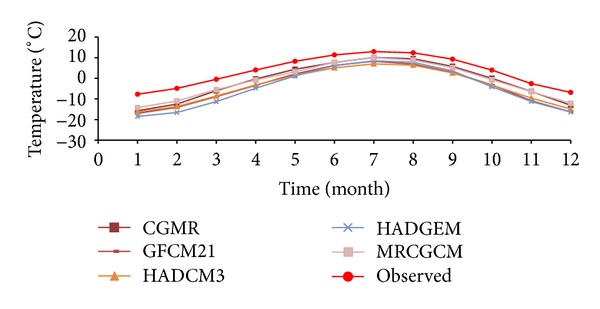
Comparison of precipitation simulated by the six best-performance models.

With regard to comparison between simulated and observed values of precipitation (Figure 6), most models have not well simulated annual changes of precipitation as the simulated values are higher than the observed. As most precipitation occurred in the period from June to September, only half of the models had higher simulated values than the observed. In general, CGMR, five patterns as CSMK3, GFCM20, GFCM21, and HADGEM, have well simulated annual precipitation trends (Figure 7), especially CSMK3, whose simulated monthly precipitation is very similar to the observation. 

**Figure 8:**
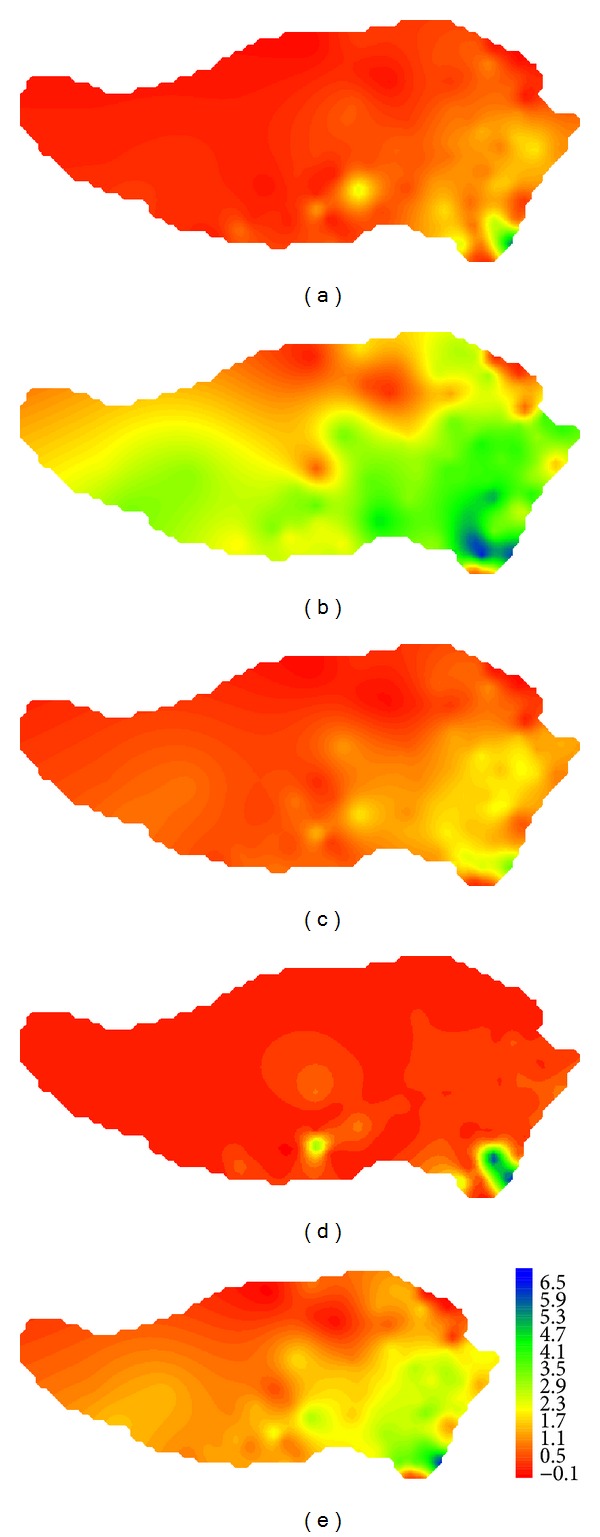
Observed values of precipitation on the Tibetan Plateau in reference period: (a) spring; (b) summer; (c) fall; (d) winter; (e) annual.

**Figure 9:**
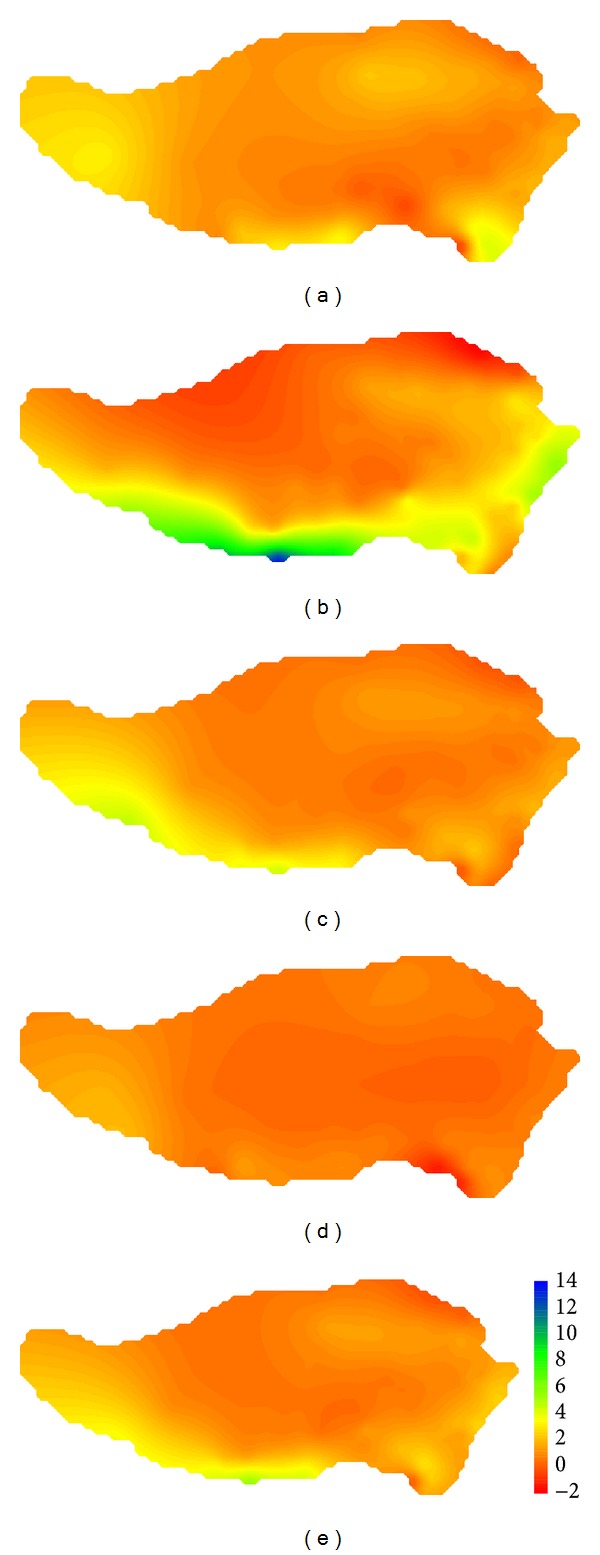
Comparison of the values of precipitation on the Tibetan Plateau in reference period between GFCM21's simulation and observation: (a) spring; (b) summer; (c) fall; (d) winter; (e) annual.

Figure 8 shows observed values of precipitation on the Tibetan Plateau in reference period which indicates that precipitation concentrated in spring and summer and there was more precipitation in southeast. Annual mean precipitation was decreasing from south to north and from east to west. As seen from differences of simulated and observed precipitations of GFCM21 shown in Figure 9, the maximum deviation occurred in summer which equaled 13.7 mm/day, followed by spring and summer. With a deviation of −1.4 ~ 2.0 mm/day, the precipitation simulation in winter is the best. For the whole region, deviation of average precipitation varied from −0.8 to 5.7 mm/day with a good simulation in the center of the region.

## 4. Projected Changes in Temperature and Precipitation

Prediction from these climate models of A1B, A2, and B1 scenarios has almost covered all possible climate changes caused by growing of greenhouse gas emissions on the Tibetan Plateau.  Table 3 presents prediction of linear trends of annual mean precipitation and temperature in the 21st century on the Tibetan Plateau under different emission scenarios. It shows that annual mean precipitation will increase for all the patterns of 3 emission scenarios with greatly various rates. There are 20 models involved under A1B scenario with a variation range of 10.3~179.8 mm/100a and an average of 92.5 mm/100a; there are 13 models involved under A2 scenario with a variation range of 32.4~186.5 mm/100a and an average of 108.7 mm/100a; there are 15 models involved under B1 scenario with a variation range of 0.3~124.3 mm/100a and an average of 61.4 mm/100a. Temperature changes of all the models have the same increasing trend as that of precipitation. Under A1B scenario, all the models except HADGEM are involved to estimate temperature changes with a variation range of 2.3~7.1°C/100a; there are 14 models involved under A2 scenario which shows the highest average increase of 5.3°C/100a and a variation range of 3.5~7.4°C/100a; 15 models are involved under B1 scenario to predict the lowest increase of 2.6°C/100a and a various range of 1.2~4.2°C/100a on the Tibetan Plateau.

## 5. Conclusions and Discussion

In order to make further climate change projections under A1B, A2, and B1 emission scenarios on the Tibetan Plateau, temperature and precipitation simulation abilities of GCMs have been evaluated which is based on the differences between simulated and observed of reference period with 22 models from IPCC AR4. Some interesting conclusions can be presented and discussed as follows.

1. 22 climate models have a certain capability to simulation temperature and precipitation on the Tibetan Plateau. The correlation coefficient of temperature of all the models (except INCM3 mode) is above 0.96, but there are still great differences in simulation performance of each model, while only GGMR, GFCM21, HADCM3, HADGEM, and MRCGCM patterns have relatively well simulated climate changes with an annual climate trend similar to the fact. Simulated precipitation of most models is higher than the observed values while the regional simulated values of some models are lower than the observed. However there are more differences between models in precipitation than in temperature. Five models namely, CGMR, CSMK3, GFCM20, GFCM21, and HADGEM have better simulated the precipitation on the Tibetan Plateau which indicates that simulation of most models need to be further improved.
2. With a general assessment of the simulation ability of temperature and precipitation, it is obvious that GFCM21 and CGMR patterns can basically reproduce climate change on the Tibetan Plateau. Take GFCM21 pattern as an example; simulated temperature in summer is more similar to the observed. At the same time, there are significant spatial differences, while there is less deviation between simulated values of temperature and precipitation and the observed with GFCM21 model in the east part of the Tibetan Plateau.
3. Under A1B, A2, and B1 emissions scenarios, there is great uncertainty in simulation of temperature and precipitation of the 21st century with climate models on the Tibetan Plateau. Temperature and precipitation of GCMs will all increase with different ratios. Precipitation under A2 scenario will increase most significantly while there is the least increase under A1B scenario. Based on evaluation of simulation performance with 22 climate models from IPCC AR4, there are great differences in the simulated values while only a few models have well simulated temperature and precipitation on the Tibetan Plateau. It is obvious that simulation with GCMs still needs to be further improved. Because of the low resolution of GCMs, parameters of physical process of climate models are also needed to be further improved, so there are great uncertainties in the simulation of temperature and precipitation. In the future, we could think of downscaling methods to predict climate changes in order to find proper method for China and provide more reliable prediction results with multi-model ensembles method. view of many uncertainties of climate models, it is of great importance to investigate climate changes in China with GCMs to reduce those uncertainties.

## Figures and Tables

**Table 1:** Information of 22 climate models of IPCC AR4 and boundary configuration on the Tibetan Plateau.

Model	Abbreviation	Resolution		Duration	Longitude range/°	Latitude range/°	Grid number
		Longitude°	Latitude/°				
BCCR_BCM2.0	BCM2	2.8125	2.79	1850–2099	74.53~105.47	25.12~41.86	33
CCCMA_CGCM3 T47 (medres)	CGMR	3.75	3.71	1850–2300	73.13~106.88	25.98~40.82	22
CNRM_CM3	CNCM3	2.8125	2.79	1860–2299	74.53~105.47	25.12~41.86	33
CSIRO_Mk3.0	CSMK3	1.875	1.8652	1871–2200	74.06~105.94	26.11~41.04	32
MIUB_ECHO-G	ECHOG	3.75	3.711	1860–2100	73.13~106.88	25.98~40.82	22
LASG_FGOALS-g1.0	FGOALS	2.8125	2.79	1850–2199	74.53~105.47	25.12~41.86	33
GFDL_CM2.0	GFCM20	2.5	2	1861–2100	75~105	26~40	37
GFDL_CM2.1	GFCM21	2.5	2	1861–2300	75~105	26~40	37
GISS_AOM	GIAOM	4	3	1850–2100	72~108	24~42	27
GISS_E-H	GIEH	5	4	1880–2099	75~105	26~40	19
GISS_E-R	GIER	5	4	1880–2300	75~105	26~40	19
UKMO_HadCM3	HADCM3	3.75	2.5	1860–2199	73.13~106.88	26.25~41.25	27
UKMO_HadGEM1	HADGEM	1.875	1.25	1860–2100	74.06~105.94	25.63~39.38	51
INM_CM3.0	INCM3	5	4	1871–2200	72.5~107.5	26~42	18
INGV_SXG 2005	INGSXG	1.125	1.1215	1870–2100	74.81~104.06	25.79~39.25	74
IPSL_CM4	IPCM4	3.75	2.535	1860–2230	73.12~103.13	25.35~38.03	29
NIES_MIROC3.2 hires	MIHR	1.125	1.1214	1900–2100	74.81~104.06	25.79~39.25	74
NIES_MIROC3.2 medres	MIMR	2.8125	2.79	1850–2300	74.53~105.47	25.12~41.86	33
MPI-M_ECHAM5-OM	MPEH5	1.88	1.87	1960–2200	74.06~105.94	26.11~41.04	32
MRI_CGCM2.3.2	MRCGCM	2.8125	2.79	1851–2300	74.53~105.47	25.12~41.86	33
NCAR_CCSM3	NCCCSM	1.40625	1.400763	1890–2099	75.23~104.77	26.61~39.22	53
NCAR_PCM	NCPCM	2.8125	2.79	1870–2099	74.53~105.47	25.12~41.86	33

**Table 2:** Comparison of temperature and precipitation between simulations of climate models and observation.

Model name for short	Simulated values		Absolute error of *T*/°C	Relative error of *P*/%	Correlation coefficient		Deterministic coefficient	
	*T*/°C	*P*/mm			*T*	*P*	*T*	*P*
BCM2	−8.2	1211.3	−11.6	146.0	0.984	0.938	0.353	0.389
CGMR	−1.4	778.8	−4.7	58.1	0.986	0.935	0.739	0.678
CNCM3	−7.4	1259.7	−10.7	155.8	0.984	0.918	0.365	0.361
CSMK3	−6.9	639.7	−10.2	29.9	0.987	0.91	0.432	0.736
ECHOG	−4.0	538.5	−7.3	9.3	0.964	0.654	0.589	−1.633
FGOAL	−1.8	592.2	−5.2	20.2	0.966	−0.084	0.649	0.559
GFCM20	−7.7	859.4	−11.1	74.5	0.982	0.923	0.365	0.623
GFCM21	−4.0	777.3	−7.3	57.8	0.985	0.922	0.570	−0.705
GIAOM	−3.1	636.2	−6.4	29.2	0.98	0.7	0.636	−3.553
GIEH	−3.1	296.9	−6.4	−39.7	0.975	−0.211	0.584	−3.069
GIER	−3.8	282.5	−7.1	−42.6	0.97	−0.141	0.572	0.490
HADCM3	−3.9	842.3	−7.3	71.0	0.984	0.881	0.541	0.644
HADGEM	−4.7	828.4	−8.0	68.2	0.982	0.94	0.545	−0.723
INCM3	−1.5	630.5	−4.8	28.0	0.942	0.579	0.650	−3.451
INGSXG	−0.1	558.4	−3.4	13.4	0.965	0.313	0.700	−2.949
IPCM4	−5.5	521.9	−8.9	6.0	0.97	0.508	0.526	−1.273
MIHR	−1.6	528.1	−4.9	7.2	0.969	0.752	0.655	−0.704
MIMR	−2.2	585.6	−5.5	18.9	0.963	0.704	0.635	−0.255
MPEH5	−2.5	596.7	−5.8	21.2	0.966	0.717	0.649	0.420
MRCGCM	−1.3	986.2	−4.6	100.3	0.986	0.904	0.732	0.282
NCCCSM	−4.7	796.0	−8.0	61.6	0.969	0.852	0.493	0.424
NCPCM	−3.2	1153.2	−6.6	134.2	0.976	0.932	0.596	0.389

(*P*: precipitation; *T*: temperature).

**Table 3:** Linear trend of temperature and precipitation simulated with models in 2000–2099.

Model	Scenario					
	Precipitation/mm·(100a)^−1^			Temperature/°C·(100a)^−1^		
	A1B	A2	B1	A1B	A2	B1
BCM2	85.2	/	64.5	3.2	3.9	1.7
CGMR	105.1	/	/	3.3	/	/
CNCM3	170.3	186.5	/	4.5	5.5	2.1
CSMK3	58.6	73.5	48.9	2.8	3.9	1.7
ECHOG	83.4	66.7	/	7.1	6.9	/
FGOALS	51.2	/	3.3	3.6	/	2.7
GFCM20	179.8	135.6	124.3	4.9	6	2.6
GFCM21	61	111.6	68.6	4.5	5.2	2.3
GIAOM	75.9	/	42.9	3	/	1.4
GIEH	52.7	/	/	3.1	/	/
GIER	/	32.4	5.5	2.3	4.5	1.4
HADCM3	118	109.1	71.6	4.6	5.5	3.2
HADGEM	/	/	/	/	/	/
INCM3	62.4	/	84.5	3.9	/	2.6
INGSXG	10.3	/	/	4.9	/	/
IPCM4	34.6	73.6	28.3	5.8	6.3	3.5
MIHR	148.5	/	67.8	6.1	/	4.2
MIMR	140	176.8	107.1	6.7	7.4	3.7
MPEH5	71.5	48.7	65	6.2	6.6	4.1
MRCGCM	127.6	132.1	76.7	3.5	4.1	2.4
NCCCSM	68.6	127.6	62.6	3.6	5.2	1.2
NCPCM	145.5	138.6	/	2.7	3.5	/

Average	92.5	108.7	61.4	4.3	5.3	2.6
